# Review: Knowledge Gained and Gaps in Understanding in the 25 Years Since Human Metapneumovirus Was First Identified as a Cause of Human Disease

**DOI:** 10.1093/infdis/jiaf187

**Published:** 2025-07-16

**Authors:** Angela R Branche, Kathryn M Edwards

**Affiliations:** Division of Infectious Diseases, Department of Medicine, University of Rochester School of Medicine, Rochester, New York; Division of Infectious Diseases, Department of Pediatrics, Vanderbilt University School of Medicine, Nashville, Tennessee, USA

**Keywords:** epidemiology and global circulation, human metapneumovirus, clinical profile of hMPV infections, risk for severe disease adults, pathogenesis and immune responses to hMPV

## Abstract

Human metapneumovirus (hMPV) is a nonsegmented, single-stranded, negative-sense RNA virus belonging to the Pneumoviridae family. It was first identified in 2001 in the nasopharyngeal secretions of 28 Dutch children with bronchiolitis collected over a 20-year period. hMPV exhibited paramyxovirus-like morphology with many genetic similarities to respiratory syncytial virus. hMPV has 1 serotype with 2 major subgroups (A and B) and 5 sublineages (A1, A2a, A2b, B1, and B2). In the wake of its discovery, a wealth of observational research has demonstrated global circulation of hMPV causing a wide spectrum of clinical disease. It accounts for 2% to 7% of all symptomatic respiratory infections in children who are universally infected by age 5 years. However, long-lasting immunity to hMPV is incomplete, and reinfections occur throughout life. With increasing age, the impact of hMPV is greater. Adult patients with hMPV infection may develop pneumonia, resulting in hospitalization and severe outcomes, such as intensive care unit admission or mechanical ventilation. Risk factors for severe hMPV are still being defined but include profound immunosuppression (20%), congestive heart failure (25%), and severe chronic obstructive pulmonary disease (20%). In this supplement, several studies from diverse geographic and clinical locations explore the pathogenesis, epidemiology, and clinical profile of hMPV as compared with respiratory syncytial virus and/or influenza and examine the impact of risk factors for severe disease, including age and chronic comorbid conditions. These data are needed to provide the basis for understanding who might benefit from future hMPV vaccines.

## Teaser

Human metapneumovirus (hMPV) is an RNA respiratory virus with global circulation. Herein we review the literature on hMPV disease burden in adults and highlight new insights presented in this supplement. These data will inform the development of future hMPV vaccines.

Human metapneumovirus (hMPV) was identified in 2001 in the nasopharyngeal secretions of 28 Dutch children with bronchiolitis collected over a 20-year period [1]. This newly discovered virus exhibited paramyxovirus-like morphology with many genetic similarities to respiratory syncytial virus (RSV) [2]. Serologic analyses of archival samples collected decades earlier in the same study demonstrated evidence of human infection for at least the 50 years prior to its identification. However, its close similarity to *Avian metapneumovirus* serotype C suggests that although hMPV is primarily a human pathogen, it likely originated from birds many centuries ago [1–3].

In the wake of its discovery, a wealth of observational research related to the epidemiology, pathogenesis, clinical disease, and host immune responses has emerged [4–9]. Clinical interest in the wider medical community, particularly related to disease in adults, has lagged behind, likely related to the absence of a unique clinical syndrome and the lower detection rates of hMPV relative to pandemic respiratory viruses that emerged in 2009 and 2019.

With approval and success of recently licensed vaccines to prevent RSV disease in adults, interest has turned to hMPV as a pathogen that may constitute a significant threat to human health and for which vaccines and other preventative measures should be developed. In this supplement, we review knowledge that has been gained in the past 2 decades and present new reports of the epidemiology and viral evolution, clinical disease, risk factors for severe disease, and host response to hMPV infections in adults. In many of the articles, we compare the impact of hMPV with other respiratory pathogens, such as influenza and RSV.

## VIROLOGY

**Figure 1.**
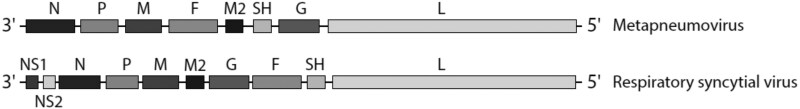
Human metapneumovirus and respiratory syncytial virus genome. Reprinted from *Feigin and Cherry's Textbook of Pediatric Infectious Diseases* with permission from JV Williams.

hMPV is a nonsegmented, single-stranded, negative-sense RNA virus belonging to the order Mononegavirales, family Pneumoviridae, and genus *Metapneumovirus* [1, 10]. Electron microscopy images of hMPV particles reveal pleomorphic spherical or filamentous particles with a lipid envelope and projections representing glycoprotein spikes [1, 11]. The original genetic analysis of hMPV by van den Hoogen et al indicated a gene order of 3′-N-P-M-F-M2(M2-1 and M2-2)-SH-G-L-5′, encoding for 9 proteins: nucleoprotein (N), phosphoprotein (P), matrix protein (M), fusion protein (F), transcription elongation factor (M2-1), RNA synthesis regulatory factor (M2-2), small hydrophobic protein (SH), glycoprotein (G), and major polymerase subunit (L) [12]. This is depicted in Figure 1, where, unlike RSV, hMPV notably lacks NS1 and NS2 proteins.

hMPV has only 1 serotype, but isolates cluster into 2 major subgroups (A and B); it also has 5 genotypes with at least 2 sublineages (A1, A2a, A2b, B1, and B2), mainly based on variations in the G protein [13, 14]. The F protein, which is synthesized as an active precursor cleaved by host proteases, is highly conserved among hMPV genotypes, but greater diversity is found in surface glycoproteins G and SH, with 59% and 37% conserved identity, respectively. Novel sublineages with diversity in the *F* and *G* genes, including duplication in the *G* gene, have also recently been described indicating continuous evolution, which is explored in this supplement [15–18].

## PATHOGENESIS AND HOST DISEASE

hMPV has been shown to infect the upper and lower respiratory tract, resulting in a wide spectrum of clinical disease [19]. Limited pathogenesis data indicate that nasopharyngeal and bronchiolar epithelial cells become infected, which may be associated with prolonged inflammation, excess mucous production, and, when infection involves the lower airways, alveolar damage [20–22].

Several studies using animal or human epithelial lung models have shown that hMPV infection induces a strong immune response characterized by type 1 interferon release that is contingent on viral replication [23]. Lymphopenia and receipt of cytotoxic therapy are risk factors for severe hMPV disease, suggesting that cellular immunity is important for hMPV clearance [24]. Polyclonal CD4+ T-cell immunodominant epitopes have been mapped to the M2-1, M, F, N, and P proteins [25, 26].

**Figure 2.**
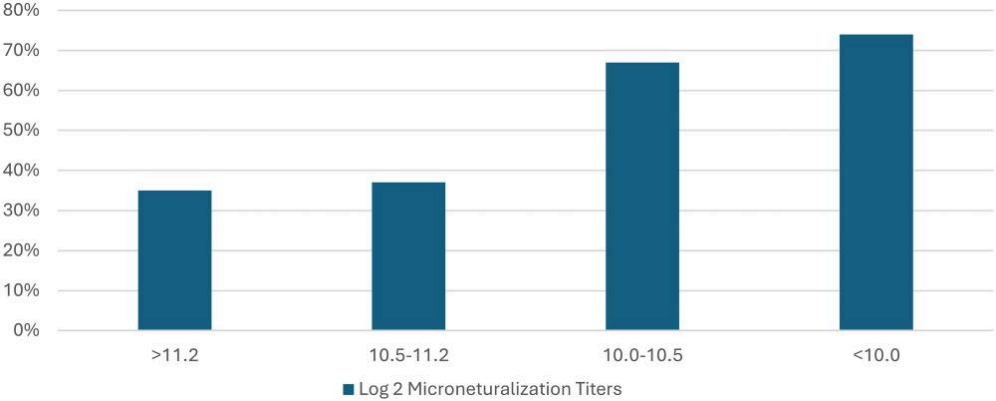
Microneutralization titers after human metapneumovirus infection in adults. Adapted from a figure in the study by Falsey et al [28].

However, long-lasting immunity to hMPV is incomplete, and reinfections occur throughout life due to waning of cellular and humoral immunity, as well as a 4-fold decrease in cross-protection across genotypes [27–31]. The primary target of neutralizing antibody is the F protein [32]. Yet, unlike RSV, where the most highly neutralizing antibodies are directed toward prefusion F, pre- and postfusion hMPV F has potent neutralizing epitopes [33, 34]. Studies have demonstrated that binding and neutralizing titers correlate with protection from hMPV infection and form the basis for hMPV vaccine development targeting humoral responses to the F protein [28] (Figure 2).

## EPIDEMIOLOGY

hMPV is a seasonal respiratory pathogen that causes infections in all age groups [1, 9, 27, 35]. It accounts for 2% to 7% of all symptomatic respiratory infections in children who are universally infected by age 5 years [7, 9, 36–40]. Reinfection then occurs throughout life, and about 2% of acute respiratory illnesses in the general adult population and 6% in community-dwelling older adults are due to hMPV [27, 41, 42].

Numerous epidemiologic studies have now documented worldwide hMPV circulation [1, 36, 39, 43–48]. Although RSV and hMPV seasons may overlap, one study published in this supplement suggests that the pattern of circulation may actually be oppositive, with hMPV appearing to circulate counterclockwise while RSV circulates clockwise on a Mercator projection [49]. The predominant subtype varies by year and likely geographic location, although multiple genotypes may cocirculate in the same season [40, 50]. However, one study in this supplement challenges the premise that only 1 subtype circulates in a given season, with phylogenetic analysis revealing an even distribution of hMPV-A and hMPV-B, with a predominance of clades A2c with a 111-nucleotide duplication and B2b over 2 overlapping seasons [51].

**Figure 3.**
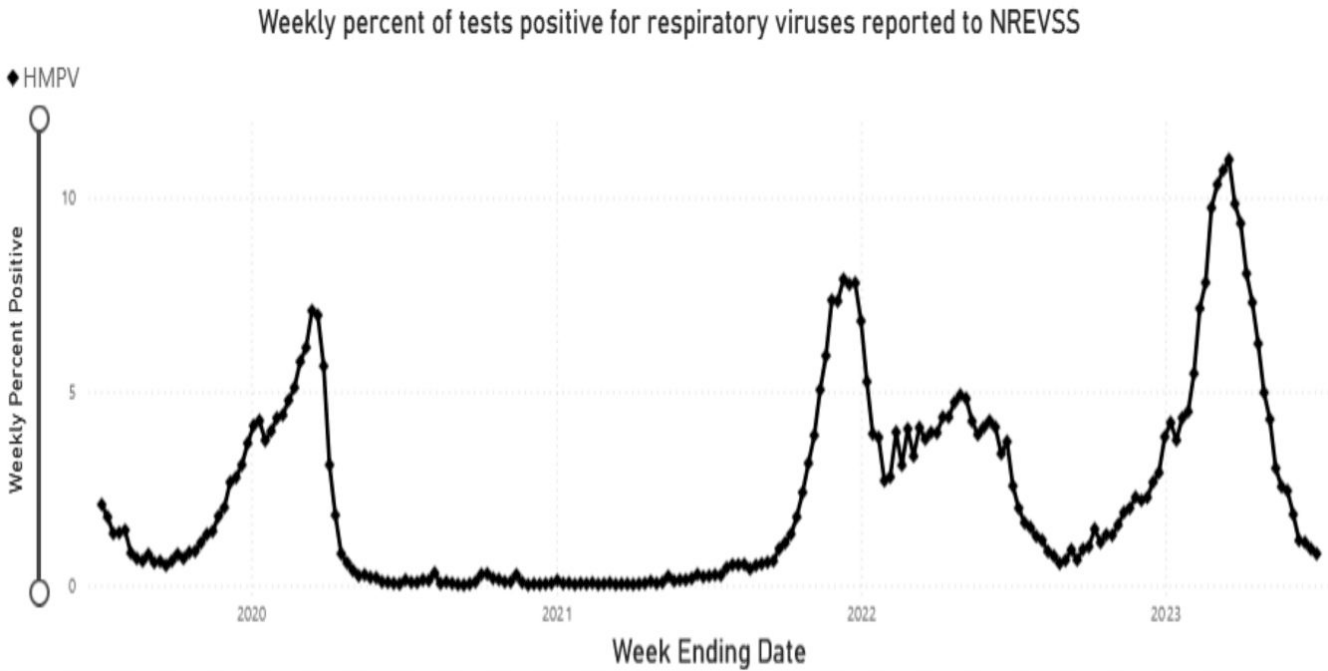
NREVSS reporting of hMPV infection: 2020–2023. Abbreviations: hMPV, human metapneumovirus; NREVSS, National Respiratory and Enteric Virus Surveillance System.

In the northern hemisphere, the virus circulates predominantly in the late winter and spring months, frequently overlapping with influenza and RSV epidemics, though generally peaking 1 to 2 months later [35, 38, 52–54]. However, hMPV activity in the northern hemisphere can also occur during the summer and early months [40, 48]. In the southern hemisphere, hMPV circulates in the summer, and in the subtropics, peak activity is in the spring and early summer [36]. In the 4 years preceding the SARS-CoV-2 pandemic, US hMPV test positivity peaked between 6.2% and 7.7% in March and April [55]. Notably, in 2021 during the COVID-19 pandemic, there was a relative absence of hMPV due to the impact of nonpharmaceutical interventions to mitigate SARS-CoV-2 transmission, as illustrated by the Seattle, Washington, investigators in this supplement [56], with a US resurgence in early fall 2022 (out of season), followed by a large outbreak late in the 2022–2023 winter season (Figure 3) [57].

In general, hMPV surveillance has lagged behind studies of influenza and RSV epidemiology. When conducted, hMPV surveillance has historically been added to studies of influenza-like illness or severe acute respiratory illness case definitions, which may not fully capture the entire burden of disease. Several articles in this supplement provide additional data on incidence and compare disease burden of hMPV with influenza and RSV. In general, these studies indicate comparable burden between RSV and hMPV but more severe burden with influenza [58]. Similar observations were made in nursing home surveillance studies [59].

## CLINICAL MANIFESTATIONS AND RISK FOR SEVERE DISEASE

Although more work is needed in this area, data support the premise that the clinical manifestations of hMPV infection in adults cover a wide spectrum of clinical presentations. Children with hMPV commonly exhibit fever, cough, sore throat, and rhinorrhea, and infection has been strongly associated with bronchiolitis, pneumonia, and croup and as a cause of recurrent wheezing episodes [36, 37, 60–64]. In contrast, adults present with more varied syndromes, ranging from asymptomatic infection and common cold symptoms, influenza-like illnesses, exacerbations of asthma, chronic obstructive pulmonary disease or congestive heart failure, and pneumonia or pneumonitis [27, 35, 65].

With increasing age, the impact of hMPV is greater. Although most previously healthy adults infected with hMPV do not require medical attention, adults with underlying cardiopulmonary conditions are at high risk for hospitalization, with illnesses primarily characterized by fever, wheezing, and severe cough. In 2 US studies of hospitalized adults, 4.5% to 8.5% of illnesses were identified as hMPV related, with those infected having high rates of chronic cardiopulmonary conditions [8, 27]. Adult patients with hMPV lower respiratory tract infection may develop pneumonia, which can progress to acute respiratory distress syndrome, and chest radiographs show patchy, multilobar infiltrates in 50% of cases [66]. Lower rates of hMPV pneumonia have been reported when compared with bacterial infection or RSV in adults [67], but one prospective observational study in this supplement describes higher rates of pneumonia in adults hospitalized with hMPV as compared with RSV [68]. Additionally, a study in this supplement, leveraging clinical data from large statewide databases, describes a higher frequency of *ICD-10* pneumonia diagnoses in adults hospitalized with hMPV when compared with adults hospitalized with either RSV or influenza and correspondingly higher rates of intensive care unit admission and mortality [69]. Risk factors for hMPV pneumonia and severe outcomes such as intensive care unit admission or mechanical ventilation are still being defined but have been described to include profound immunosuppression—particularly, in cases of solid organ or stem cell transplant (20%), congestive heart failure (25%), and severe chronic obstructive pulmonary disease (20%) [70].

In this supplement, several studies from diverse geographic and clinical locations explore the comparative profile of hMPV clinical manifestations with patients diagnosed with RSV and/or influenza. They also examine whether hMPV disease severity is affected by the same risk factors for severe disease previously described for other viruses, including age and chronic comorbid conditions. Two studies published in this supplement confirm that, like RSV, advanced age and comorbid conditions increase the frequency of hMPV-associated hospitalization and the severity of infection and introduce frailty as a potential important risk factor [71, 72].

## DIAGNOSIS

Because identification of hMPV with cell culture is difficult and serology is useful only in a research setting, molecular techniques have been adopted as the optimal approach to diagnosis [73, 74]. hMPV detection is now incorporated into several commercially available multiplex polymerase chain reaction assays that are typically performed on nasal or nasopharyngeal swab samples [75]. But lower respiratory tract samples such as bronchoalveolar lavage fluid and sputum can also be used, with several reports indicating a higher yield for hMPV detection in lower respiratory tract samples as compared with nasal swabs [76, 77]. However, most medical institutions employ, as standard of care, triplex assays that include influenza, RSV, and SARS-CoV-2, whereas hMPV testing requires the availability of an assay with a wider panel of viruses that is also more costly. Consequently, hMPV diagnosis in the outpatient or inpatient testing is infrequent, which has impeded a better understanding of the clinical impact of hMPV infection and risk factors for severe disease outcomes in adult populations. Most of the studies in this supplement use molecular testing. Yet, similar to what has been found with RSV, the addition of serologic testing as well as molecular testing of multiple other sample types in future prospective studies may increase the yield of detection and improve our understanding of disease burden [78, 79].

## TREATMENT AND PREVENTION

While there are no approved treatments for hMPV infection, a better understanding of the pathogenesis of disease may present potential targets for the development of antiviral therapies. For example, one study in this supplement assessed host responses to infection using novel gene expression methodologies, which may help elucidate mechanisms of disease. Notably, genes with higher expression in individuals infected with hMPV were associated with antigen binding, immunoglobulin production, and adaptive immunity, while genes increased in RSV infection were associated with natural killer T cells [68].

### Vaccine and Monoclonal Antibody Research

**Figure 4.**
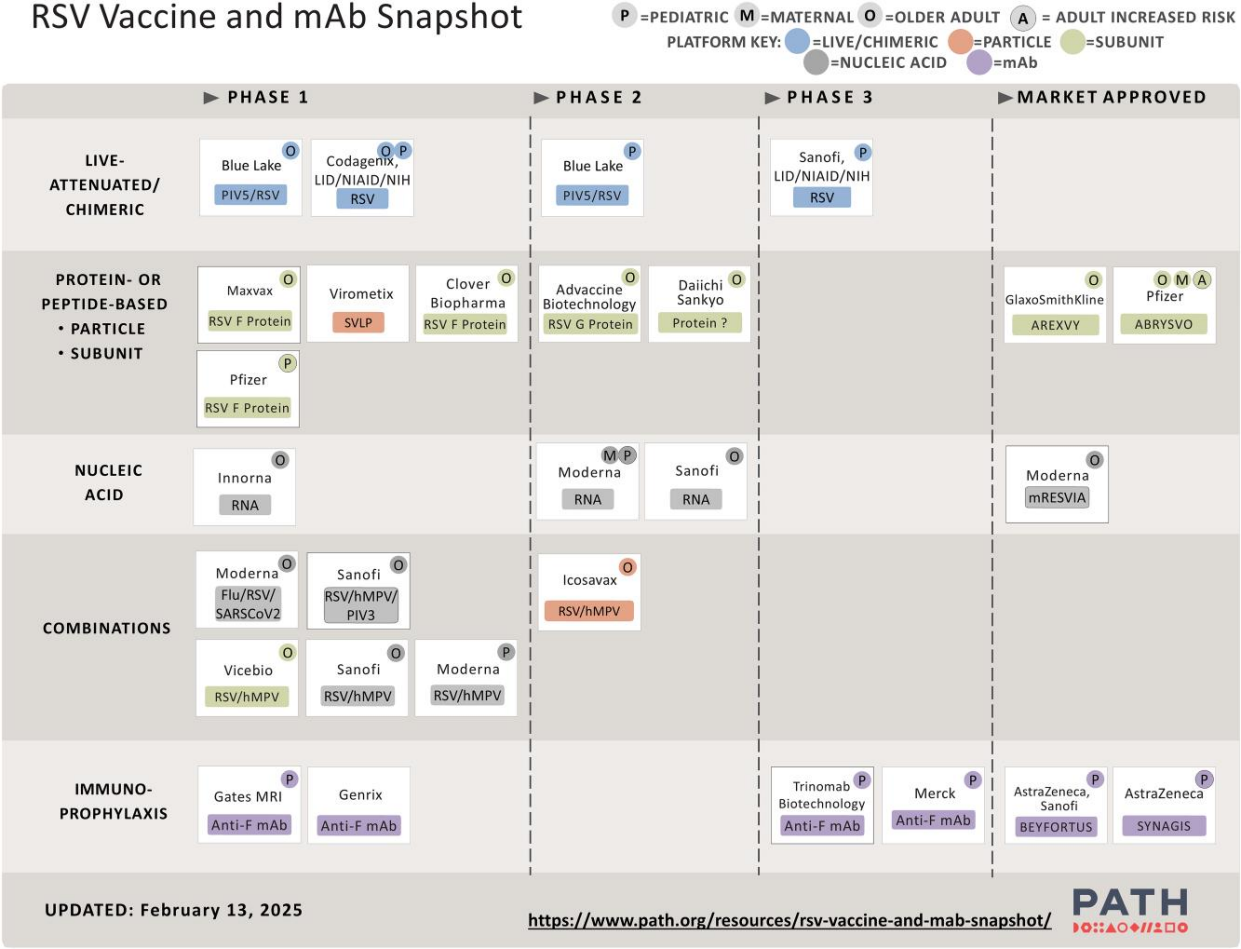
PATH RSV vaccine and mAb snapshot: February 2025. Abbreviations: mAb, monoclonal antibody; RSV, respiratory syncytial virus.

In contrast to the treatment landscape, vaccine research and development for prevention of hMPV infection and disease in adults have been active in the last decade. This has likely been stimulated by the high efficacy of the pre-F RSV vaccines licensed in 2023 and 2024 for use in adults and the reduction in RSV-confirmed disease since licensure [80–82]. For example, a phase 1 dose-ranging trial tested an mRNA platform vaccine encoding the sequences for the full-length fusion proteins of hMPV and PIV3 coformulated in a lipid nanoparticle [83]. Geometric mean titers in vaccine recipients were similar across doses and 6-fold higher than baseline against hMPV-A and hMPV-B subtypes. However, a recent age de-escalation phase 1 clinical trial of a combination hMPV/RSV mRNA vaccine in young children was halted due to concerns for immune-enhanced RSV and hMPV disease [84]. Studies are ongoing in adults with several hMPV vaccines utilizing mRNA and other platforms, including combination vaccines against multiple viral pathogens (Figure 4) [85].

Similarly, the development of monoclonal antibodies against hMPV has been accelerated as a result of the successes of RSV monoclonal antibodies for the prevention of disease in infants. However, despite the similarities between the RSV and hMPV F protein, targets sites that provide the most potent neutralization activity differ between the viruses [86]. Moreover, although hMPV anti-F monoclonal antibodies have shown neutralization activity in animal models, a better understanding of the mechanism of neutralization is still needed, as well as data on the potential target population [86].

## FUTURE DIRECTIONS FOR HMPV RESEARCH

Since its discovery in 2001, hMPV has gained recognition as a significant cause of acute respiratory illnesses in children and adults [4–9]. Despite challenges in defining the true burden of disease—due to low clinical suspicion and the limited availability and nonuniform usage of multiplex molecular diagnostics in inpatient and outpatient settings—observational studies suggest that the burden of hMPV is similar to that of RSV, with clinical disease manifestations similar to RSV and influenza [19]. Work in this supplement offers critical information on the epidemiology and viral evolution of hMPV before and after the COVID-19 pandemic, the incidence of severe disease with hMPV, and cofactors that may increase risk of hMPV hospitalization in adults. Moreover, investigators explore important comparisons of hMPV infection in adults with that caused by RSV or influenza, which are needed to provide the basis for understanding whether there is a true need to develop hMPV vaccines and who might benefit most from vaccination. Notably, the seasonality of hMPV circulation and the groups at risk for severe disease may differ from those of RSV or influenza and affect future vaccine policy. Thus, although the body of literature related to hMPV infection in adults continues to expand, large prospective population-based studies, with systemic testing from geographically diverse regions to assess incidence, pathogenesis, and clinical disease, are likely still needed. Moreover, data are needed on the rate and impact of viral and bacterial coinfections. Such data may also result from analysis of cases seen in large placebo-controlled efficacy trials of hMPV vaccines in older adults.

## Notes

***Disclaimer.*** All works presented were created independently by the authors with no input that might constitute a conflict of interest from the funder.

***Supplement sponsorship.*** This article appears as part of the supplement “Human Metapneumovirus Disease Burden in Older Adults and Implications for Adult Vaccination Strategies,” sponsored by AstraZeneca.

***Financial support.*** The collection or analysis of some of the data presented in this supplement was largely supported by grant funding from AstraZeneca.
